# Predicting disease progression under a “treatment-naive” counterfactual scenario in a competing risks framework

**DOI:** 10.3389/fepid.2026.1850482

**Published:** 2026-09-15

**Authors:** Daniela Zugna, Nicolas Destefanis, Renata Zelic, Andreas Pettersson, Lorenzo Richiardi

**Affiliations:** 1 Department of Medical Sciences, Cancer Epidemiology Unit, University of Turin, Turin, Italy; 2 Department of Molecular Medicine and Surgery, Karolinska Institute, Stockholm, Sweden; 3 Department of Pelvic Cancer, Cancer Theme, Karolinska University Hospital, Stockholm, Sweden; 4 Department of Medicine Solna, Clinical Epidemiology Division, Karolinska Institute, Stockholm, Sweden

**Keywords:** causal inference, competing risks, counterfactual risk, g-formula, prognostic model

## Abstract

**Purpose:**

Prognostic models aim to estimate the individual risk of developing a specific health outcome based on patient characteristics and prognostic factors. A key factor affecting performance over time and across different populations is evolving treatment strategies. These shifts can alter the relationship between predictors and outcome, as well as occurrence of competing events. Counterfactual risk models may yield more reliable estimates across different time periods and treatment regimens than traditional approaches like ignoring treatment, restricting analysis to untreated patients, stratifying, or adjusting for treatment.

**Patients and methods:**

In the Turin Prostate Cancer Prognostication cohort study, which includes patients diagnosed with non-metastatic prostate cancer and followed longitudinally, the performance of Memorial Sloan Kettering Cancer Center prediction model was evaluated in predicting metastatic progression up to 5 years after diagnosis in the presence of competing mortality risk. Using the g-formula, time to the two competing events was generated under a counterfactual scenario in which no patients received prostatectomy. The performance of the MSKCC model developed in the TPCP cohort and applied to the counterfactual cohort (applied model) and the model updated under this counterfactual scenario (updated model) were compared.

**Results:**

At 5 years post-diagnosis, the updated model had an Area Under the Curve (AUC) of 0.84 (95% CI: 0.82; 0.85) and the applied model had an AUC of 0.83 (95% CI: 0.81, 0.85). The calibration slope for the updated model was 1.02 (95% CI: 0.99, 1.12) compared to 1.07 (95% CI: 0.97, 1.17) for the applied model. Calibration intercepts were 0.00 (95% CI: −0.10, 0.10) and 0.05 (95% CI: −0.04, 0.15) respectively. The predicted probabilities of the outcome of interest under counterfactual treatment strategy varied not only because of the treatment itself, but also due to its impact on competing risks.

**Conclusion:**

When a prediction model cannot be validated in a new population due to treatment shifts, counterfactual risks under hypothetical regimens can still be cautiously estimated. In particular, counterfactual modeling should be considered as a useful complementary assessment of model transportability and calibration under clinically relevant treatment scenarios.

## Introduction

Prognostic models aim to predict individual risks of developing a specific health outcome based on measurable characteristics. They can inform both patients and clinicians about various aspects of the decision-making process, particularly regarding treatment options (1). The development and applicability of a prognostic model involve two main phases: model development, which includes model specification and predictor selection, and model validation (internal or/and external), which provides reliable estimates of model performance and thus allows for an evaluation of model generalizability to specific settings (2). The performance of a prediction model may vary depending on the population to which it is applied or the period during which it is used.

One key factor influencing external validity and performance of prognostic models across time and populations is the evolution of treatment strategies. Changes in treatment strategies can alter the associations between the predictors and the outcome of interest, as well as affect competing risks (3). Ideally, a prognostic model should be developed and validated in treatment-naïve patients to guide the individual treatment decisions. However, in clinical practice, patients typically receive treatment based on their baseline characteristics, the corresponding predicted (or perceived) risk of developing the outcome of interest, and their life expectancy. Therefore, the performance of the prognostic models may be masked by the efficacy of the treatment, especially when clinical decision making is based on the prognostic characteristics included in the model. In particular, the predicted risk of the outcome in treated patients may underestimate their actual risk in the absence of treatment. As a result, the use of a prognostic model in clinical practice might lead to an underestimation of the risk and, consequently, to the potential undertreatment of precisely those patients who would have benefited the most from the therapy.

Several approaches have been adopted to account for treatment in prognostic models (4–6). One common approach is to ignore treatment altogether. As described above, when the treatment influences the outcome of interest, this strategy can be suboptimal. Another strategy is to restrict the analyses to untreated patients. However this may introduce selection bias, as treatment decisions are often based on patient characteristics and/or other prognostic factors. As a result, untreated individuals may have a relatively higher/lower probability of the outcome, which can lead to an overestimation/underestimation of risk when the model is applied to all patients. Additionally, untreated patients may exhibit a broader range of outcome probabilities compared to treated patients, thus translating in a lack of information for some of the patients. A third approach involves modelling treatment directly, for example by stratifying by treatment group or including treatment as a covariate in the regression model. Yet, this can be challenging when treatment regimens evolve over time or when treatment effects are modified by individual characteristics. Moreover, prognostic models are typically used to inform pre-treatment decision making and thus information on the treatment is often lacking when models are used in the clinical setting.

These limitations can be addressed by posing a counterfactual question during model development: what would happen if one could intervene on the treatment assignment? For instance, based on baseline predictors, one could estimate the risk of an outcome at a specific time point (e.g., 5 years after diagnosis) under various hypothetical scenarios, such as if all, none, or a certain proportion of the patients had received a particular treatment. In this context, counterfactual risk prediction models can offer more generalizable results over time and enable risk prediction in settings with different treatment regimens (7, 8).

In this study, we used data from Turin Prostate Cancer Prognostication (TPCP) cohort study, which includes patients diagnosed with non-metastatic prostate cancer (PCa) and followed-up for several years since diagnosis (9). Given the wide variation in PCa outcomes, accurate risk stratification is crucial for treatment decision-making. Several pre-treatment risk stratification tools have been developed to classify patients based on their estimated risk of biochemical recurrence or lethal disease (i.e., metastatic disease or death from prostate cancer), using clinical and pathological parameters collected at diagnosis. Based on these risk estimates, clinicians can determine the most appropriate primary treatment strategy, which may include deferred treatment (active surveillance or, in the past, watchful waiting), radical treatment (radical prostatectomy or radiation therapy), primary androgen deprivation therapy, or palliative treatment. The predictive performance of the Memorial Sloan Kettering Cancer Center (MSKCC) prediction model was assessed and recently updated using TPCP data, in predicting metastatic progression under the observed treatment regimen (https://www.mskcc.org) (10–12). The aim of this study was to compare the performance of the MSKCC prediction model developed in the TPCP cohort with that of the MSKCC prediction model updated in a counterfactual cohort in which no patients received prostatectomy, for predicting metastatic progression in the presence of competing mortality risk. Prostatectomy was selected a-priori as the treatment of interest because it is the main radical treatment with curative intent for localized prostate cancer and therefore represents a clinically relevant intervention for illustrating our proposed methodology.

## Material and methods

### Data sources

The TPCP cohort includes consecutive patients diagnosed with prostate cancer from 1st January 2008 to 31st December 2013, whose prostate biopsy was evaluated at one of the two Pathology wards of the University Hospital “Città della Salute e della Scienza di Torino”, in Turin, Italy. Details of the study cohort have been described previously (9). To be eligible for inclusion, patients had to be under 85 years of age, residents in the Province of Turin, and without systemic metastases at diagnosis. Each patient was followed from the date of the diagnostic biopsy until the date of death, emigration outside the Province of Turin, or 31st December 2021, whichever occurred first. The primary outcome of the TCPC study was the development of metastatic PCa, defined as the first record of metastatic disease after diagnosis. Clinical and histopathological data were obtained for each patient. Post-diagnosis information on re-biopsies treatments, including TURP (transurethral resection of the prostate), radiotherapy, hormonotherapy, and prostatectomy, was also collected, along with the corresponding dates of first visit, prescription drugs and surgery. For the sake of this paper we defined as treated patients those who underwent radical prostatectomy in the first six months after diagnosis and as untreated those who were handled otherwise.

The study was approved by the Local inter-Hospital Ethical Committee “A.O. U. Città della Salute e della Scienza di Torino, AO Ordine Mauriziano di Torino, ASL Città di Torino” (N. 595/2020).

### Statistical analysis

Assuming the missingness process is missing at random (MAR) or missing not at random (MNAR), depending only on the covariates and not on the outcome, a complete case analysis was performed (13).

The overall aim of the study was to assess whether a prognostic model developed in the observed cohort while ignoring treatment remains valid under a hypothetical no-treatment scenario, representing the target population for individualised risk prediction. To address this question, we compared model performance across the analytical steps summarized in Table 1.

**Table 1:** Analytical steps to compare model performance under several scenarios.

Step	Description	Methodological question addressed
1. MSKCC prediction model developed in the observed cohort	The MSKCC prediction model is updated in the observed cohort while ignoring treatment information.	How does a prognostic model perform when developed from observed data without accounting for treatment, as commonly done in practice?
2: MSKCC prediction model evaluated in untreated patients	The updated prediction model developed in the observed cohort is applied only among patients who did not undergo prostatectomy.	Can restricting validation to untreated patients provide an adequate approximation of model performance under a treatment-naïve scenario?
3: MSKCC prediction model applied to the counterfactual cohort (*applied model*)	The prediction model developed in the observed cohort is applied to the simulated cohort generated under the hypothetical intervention in which no patient receives prostatectomy.	Does a prognostic model developed in the observed cohort remain valid in the hypothetical treatment-naïve population?
4: MSKCC prediction model updated in the counterfactual cohort (*updated mode*l)	The MSKCC prediction model is re-estimated using the simulated counterfactual treatment-naïve cohort.	What predictive performance could be achieved if the prognostic model was developed directly in the hypothetical treatment-naïve population?

### MSKCC prediction model in the observed cohort

We updated the MSKCC prediction model in the observed cohort using the conventional approach of ignoring treatment information. To account for competing events, we fitted two separate cause-specific Cox proportional hazards models, one for metastatic disease and one for death from other causes (14, 15), after testing the proportional hazards assumption using Schoenfeld residuals. The model for metastatic PCa included all prognostic variables from the MSKCC prediction model: age at diagnosis, prostate-specific antigen (PSA), the International Society of Urological Pathology (ISUP) histological grade, clinical tumor stage (cT1, cT2 and cT3), and the number of positive and negative biopsy cores. The model for death from other causes included the same covariates as the metastatic PCa model, with the addition of year of diagnosis, Charlson-Romano comorbidity index and socio-economic status, based on a deprivation index calculated at census block level (16, 17). Linearity of continuous variables was assessed by comparing models including linear term with models including restricted cubic splines using likelihood ratio tests. Treatment-by-covariate interaction terms were tested, but no evidence of effect modification was found. Internal validation was carried out by bootstrap resampling (1). Model performance was assessed by Area under the Curve (AUC), calibration intercept and slope at several time points and the calibration plot at 5 years since diagnosis (1, 18, 19). Time at risk was defined as the period from diagnosis until the occurrence of metastatic PCa, death from other causes, or 5 years since diagnosis, whichever came first. The last follow-up was truncated at 5 years to minimize the treatment-related changes beyond the early post-diagnosis period.

### Counterfactual cohort simulation

We used the parametric g-formula to estimate the metastatic PCa risk under the hypothetical scenario in which no patients received prostatectomy. This approach allows for consistent estimation of outcome probabilities under hypothetical treatment assignment, provided that all relevant predictors of both treatment and outcome are measured (20, 21). In our context, the decisions to undergo prostatectomy are typically made at the time of diagnosis, based on tumour severity and patient characteristics. We assumed that this decision remained unchanged during the 6 months following diagnosis. That is, if a patient underwent surgery within 6 months, the decision to do so was considered to have been made at diagnosis. Under this assumption, there were no potential time-dependent confounders affecting the treatment-outcome association. To generate the counterfactual scenario we followed these steps: (i) we divided the 5-year follow-up period into discrete time intervals (monthly); (ii) in each interval, we estimated the probability of metastatic PCa and death from other causes using pooled logistic regression, conditioning on treatment status in the current and the previous interval, as well as on the prognostic variables from the MSKCC prediction model; (iii) we estimated the probability of initiating prostatectomy, modelled as a time-dependent variable. Patients were considered untreated from diagnosis until the date of prostatectomy and treated thereafter, with treatment status defined as an absorbing state. This was modelled using logistic regression, with the MSKCC prognostic variables, year of diagnosis, number of comorbidities at diagnosis, and socio-economic status included as predictors. Finally, (iv) using Monte Carlo simulations we generated a dataset reflecting the counterfactual scenario in which no patients received prostatectomy. Outcomes were assigned based on the probabilities estimated in step ii, using each patient's baseline covariates and the corresponding hypothetical treatment assignment. Although the treatment model was estimated as part of the standard g-formula implementation, under the deterministic intervention of no prostatectomy, treatment was fixed to 0 throughout follow-up when simulating the counterfactual outcomes. The simulated dataset was generated at ten times the size of the observed cohort to reduce Monte-Carlo error and provide a more precise numerical approximation of the estimated data-generating distribution.

### Applied and updated prediction models in the counterfactual cohort

The MSKCC prediction model developed in the observed cohort was applied to (hereafter, *applied model*) and updated (hereafter, *updated model*) in this counterfactual cohort to assess its robustness under a no-treatment scenario. We compared the performance of the applied and updated prediction models by Area under the Curve (AUC), calibration intercept and slope at several time points and the calibration plot at 5 years since diagnosis. Confidence intervals for regression coefficients and model performance were obtained by bootstrap resampling of the simulated cohort and therefore reflect uncertainty conditional on the estimated g-formula, i.e., they capture uncertainty about the prognostic model parameters and not to the causal data-generating model. Similarly, internal validation was performed by bootstrap resampling of the simulated cohort and therefore reflects optimism of the prognostic model conditional on the estimated g-formula. Performance measures of the applied model were not corrected for optimism, as the model was evaluated on data different from the development dataset. However, the simulated data were not independent of the observed data, hence this analysis cannot be interpreted as external validation but rather as an assessment of model transportability under a hypothetical no-treatment intervention. Finally we compared the predicted risk of metastatic PCa in the observed cohort by using the applied and updated models.

Analyses were carried out using the statistical software R (https://www.R-project.org/); the *g-formula* package was used to implement the g-formula (22, 23).

## Results

The observed cohort includes 891 patients. Since a case-complete analysis was performed, 181 patients (20%) with at least one missing value in the predictors or in treatment regimen were excluded (Supplementary Appendix Table 1). The baseline characteristics of the 710 patients included in the analysis are reported in Table 2. Of these, 302 (42.5%) underwent radical prostatectomy within 6 months of diagnosis. The median time from diagnosis to treatment was 78 days, with an interquartile range from 58 to 103 days. During the 5-year follow-up, 48 patients developed metastatic PCa and 83 died from other causes. One patient was lost to follow-up and was excluded from further analysis for simplicity. Non-parametric cumulative incidences for the two competing events are shown in Figure 1.

**Table 2:** Descriptive characteristics of the patients of the TPCP cohort included into the analysis (*N* = 710).

Variable	*N* median	% IQR
Age at diagnosis (year), median (IQR)	70	(64-74)
Calendar year at diagnosis		
2008	207	29.1
2009	146	20.6
2010	58	8.2
2011	107	15.1
2012	100	14.1
2013	92	13.0
PSA (ng/mL), median (IQR)	6.7	(5.1-10.2)
Clinical tumour stage		
Ct1c	312	43.9
cT2	249	35.1
cT3	149	21.0
ISUP grade		
1	84	11.8
2	226	31.8
3	136	19.1
4	195	27.5
5	69	9.7
Number of positive cores, median (IQR)	3	2-5
Number of negative cores, median (IQR)	11	8-14
Number of comorbidities at diagnosis		
0	603	84.9
1	31	4.4
2+	76	10.7
Social deprivation index, median (IQR)	0.63	(−0.48-1.46)
Treatment (within 6 months)		
Prostatectomy	302	42.5
Other (radiotherapy, ormonotherapy, palliative therapy)	408	57.5

IQR, interquartile range; PSA, prostate-specific antigen; ISUP, international society of urological pathology.

**Figure 1:**
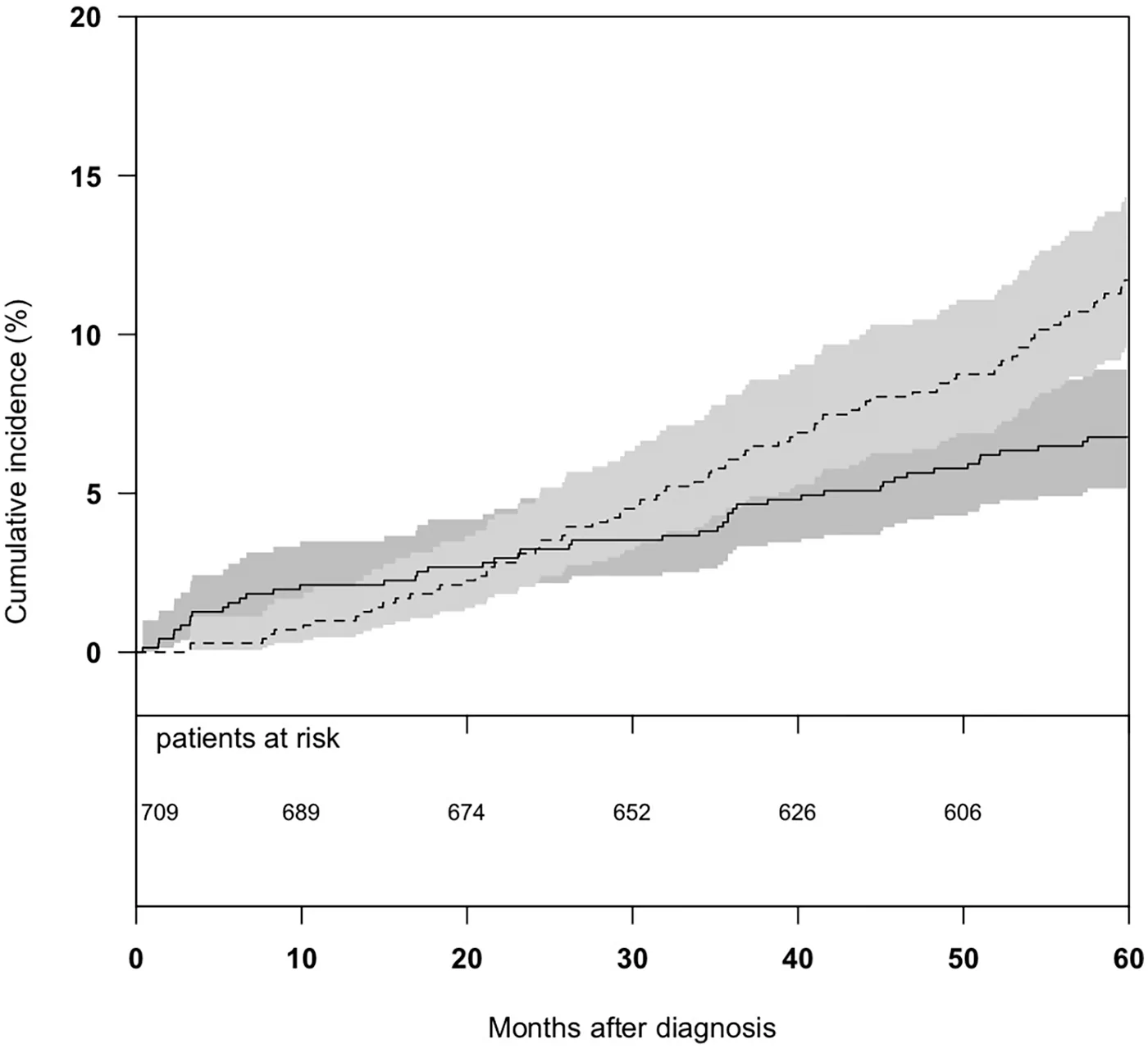
Non-parametric cumulative incidence curves for metastatic prostate cancer (*N* = 48, solid line) and death from other causes (*N* = 83, dash line) with their 95% confidence interval.

The proportional hazards assumption was satisfied for both outcomes (*p* = 0.51 for metastatic disease and *p* = 0.56 for death from other causes). Linearity assumptions for continuous variables was also satisfied (*p* = 0.14 and *p* = 0.32 for PSA in the models for metastatic PCa and death from other causes respectively, *p* = 0.12 for socio-economic status in the model for death from other causes). Coefficients of the prognostic models for metastatic PCa and death from other causes developed in the cohort are reported in the Supplementary Appendix Tables 2a,b. In terms of discrimination, the MSKCC prediction model discriminated well in the cohort, with an AUC of 0.87 (95% CI: 0.79, 0.92) at 3 years, 0.83 (95% CI: 0.76, 0.89) at 4 years and 0.82 (95% CI: 0.75, 0.89) at 5 years since diagnosis. The optimism ranged from 0.00 to 0.03 with a median of 0.02 (Supplementary Appendix Figure 1). Regarding calibration, at 3, 4 and 5 years since diagnosis, the calibration intercept values were 0.15 (95% CI: −0.24, 0.55), 0.01 (95% CI: −0.35, 0.38) and 0.00 (95% CI: −0.34, 0.33), respectively, with calibration slopes of 1.26 (95% CI: 0.75, 1.78), 1.04 (95% CI: 0.66, 1.41) and 1.01 (95% CI: 0.68, 1.33), respectively. The optimism ranged from 0.07 to 0.13 with a median of 0.09 for the calibration intercept and from 0.12 to 0.21 with a median of 0.18 for the calibration slope. Calibration plot at 5 years since diagnosis is shown in Supplementary Appendix Figure 2. When the model developed in the observed cohort was applied in patients who did not undergo prostatectomy (out of 408 patients, 34 metastatic progressions and 70 deaths from other causes), the predicted risk of metastatic progression was underestimated as indicated by the calibration intercept and slope values (Supplementary Appendix Table 3).

The counterfactual dataset, based on the scenario in which no patients underwent prostatectomy, maintained the same distribution of baseline predictors, while time to the main outcome or competing events were simulated accordingly. A dataset of 7,090 observations was generated using Monte-Carlo methods to balance accuracy and computational complexity while maintaining the possibility of comparison of the number of events with the observed dataset of 709 observations. Under this hypothetical scenario, 523 patients developed metastatic disease, while 1,059 patients died from other causes. These numbers are explained by the association between prostatectomy and competing risks. When prostatectomy was included in the regression models fitted to the observed data, the estimated hazard ratios suggested a lower risk of metastatic PCA, although confidence intervals were wide and crossed the null (HR = 0.64, 95% CI: 0.32, 1.29) and, mainly, a lower risk of death from other causes (HR = 0.39, 95% CI: 0.20, 0.75). Hence, in the scenario of absence of prostatectomy, the risk of death from other causes increased limiting the occurrence of metastatic cancer. Coefficients of the updated models for each of the two outcomes are shown in the Supplementary Appendix Tables 4a,b. The updated prediction model showed similar discrimination to the applied prediction model (Figure 2). At 5 years post-diagnosis, the updated model had an AUC of 0.84 (95% CI: 0.82, 0.85) and the applied model had a AUC of 0.83 (95% CI: 0.81, 0.85). The optimism ranged from 0.003 to 0.014 with a median of 0.004 for the updated model. The calibration intercept and slope for the two models are shown in Figure 3. At 5 years since diagnosis, the calibration slope for the updated model was 1.02 (95% CI: 0.99, 1.12) compared to 1.07 (95% CI: 0.97, 1.17) for the applied model, while the calibration intercept was 0.00 (95% CI: −0.10, 0.10) for the updated model compared to 0.05 (95% CI: −0.04, 0.15) for the applied model. The optimism ranged from 0.002 to 0.020 with a median of 0.009 for the calibration slope and from −0.005 to 0.018 with a median of 0.006 for the calibration intercept in the updated model. Calibration plot at 5 years since diagnosis for both models is shown in Supplementary Appendix Figure 3. Performance measures of the fitted models at 5 years since diagnosis are summarised in Supplementary Appendix Table 5.

**Figure 2:**
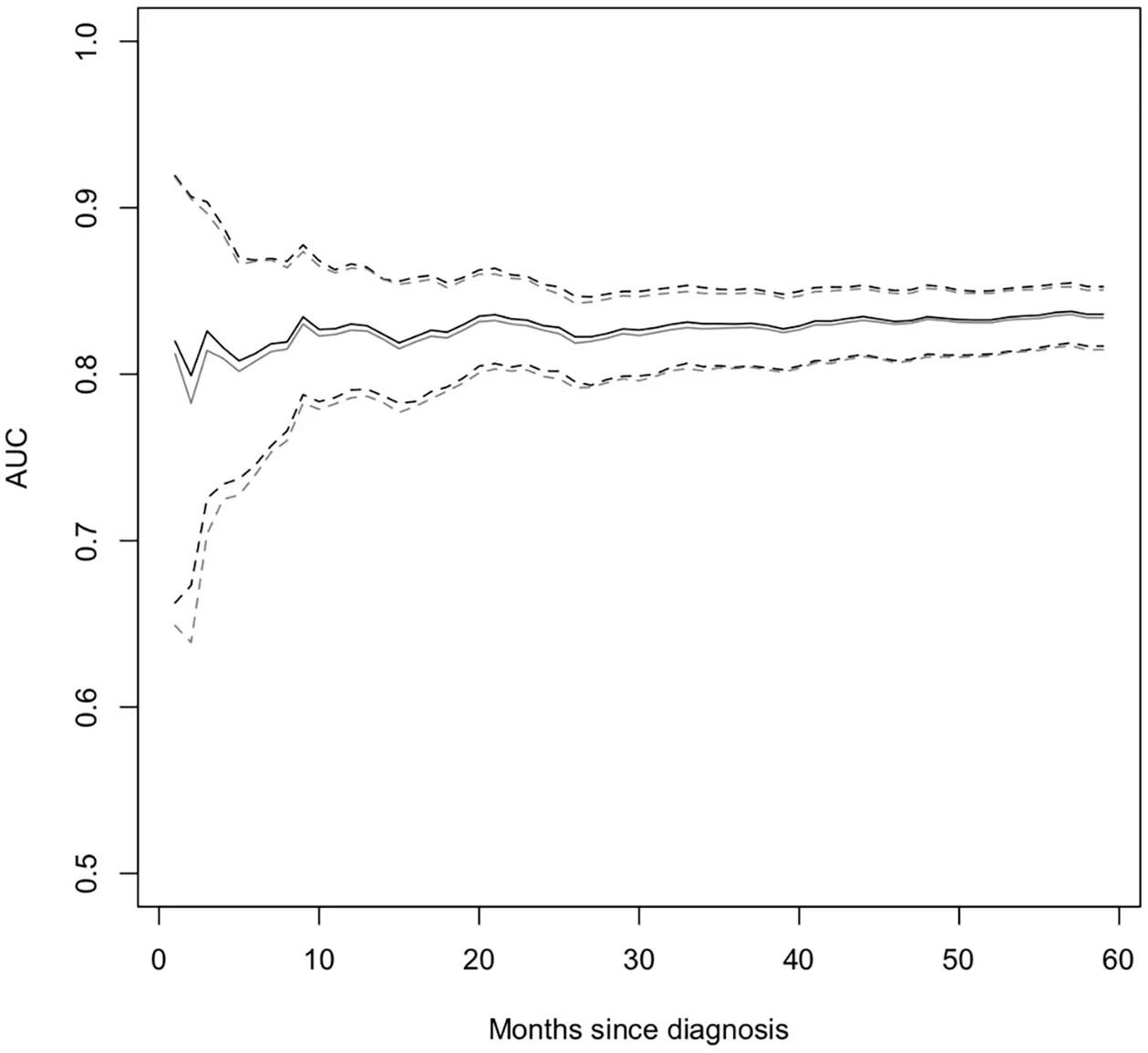
Area under the curve (AUC) of the MSKCC prediction model updated in the counterfactual cohort where nobody underwent treatment (black line) and developed in the observed cohort and applied in the counterfactual cohort (gray line) with 95% confidence intervals (dash lines).

**Figure 3:**
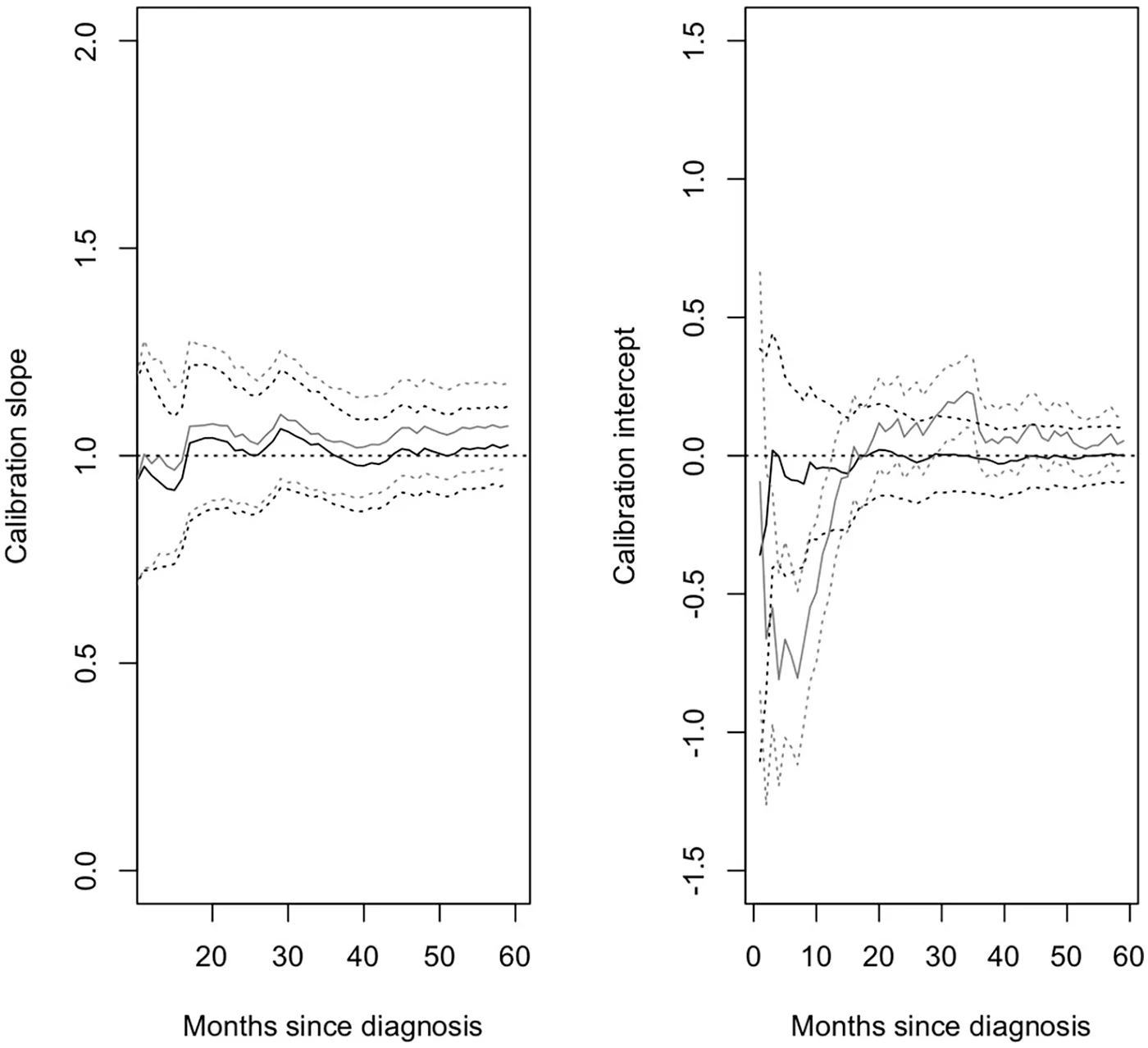
Calibration slope and calibration intercept of the MSKCC prediction model updated in the counterfactual cohort where nobody underwent treatment (black line) and developed in the observed cohort and applied in the counterfactual cohort (gray line) with 95% confidence intervals (dash lines).

The predicted 5-year metastatic PCa risks in the observed cohort from the MSKCC prediction model developed in the observed cohort and the updated prediction model under the hypothetical scenario in which no patients received prostatectomy are compared in Table 3 using 5%-width risk intervals. The updated model led to risk reclassification of the MSKCC prediction model in 11.6% patients. Specifically 7.1% had a higher predicted risk and 4.5% had a lower predicted risk under the updated model compared to the MSKCC prediction model. Interestingly the updated model predicted a higher risk of death from other causes in 53.2% patients compared to the MSKCC prediction model (Supplementary Appendix Table 6).

**Table 3:** Reclassification table of the predicted risks of progression to metastasis at 5 years since diagnosis in the observed cohort (*N* = 709) by the MSKCC prediction model developed in the observed cohort (applied model) and the MSKCC prediction model updated under the counterfactual scenario in which no patient was treated (updated model).

Applied model	Updated model												
	(0%–5%)	(5%–10%)	(10%–15%)	(15%–20%)	(20%–25%)	(25%–30%)	(30%–35%)	(35%–40%)	(40%–45%)	(45%–50%)	(50%–60%)	(60%–70%)	(70%–100%)
(0%–5%)	466	13	0	0	0	0	0	0	0	0	0	0	0
(5%–10%)	5	82	6	0	0	0	0	0	0	0	0	0	0
(10%–15%)	0	10	30	10	0	0	0	0	0	0	0	0	0
(15%–20%)	0	0	7	17	3	0	0	0	0	0	0	0	0
(20%–25%)	0	0	0	4	9	3	0	0	0	0	0	0	0
(25%–30%)	0	0	0	0	2	3	2	0	0	0	0	0	0
(30%–35%)	0	0	0	0	0	1	4	1	0	0	0	0	0
(35%–40%)	0	0	0	0	0	0	2	4	2	2	0	0	0
(40%–45%)	0	0	0	0	0	0	1	0	3	1	2	1	0
(45%–50%)	0	0	0	0	0	0	0	0	0	3	1	0	0
(50%–60%)	0	0	0	0	0	0	0	0	0	0	4	2	0
(60%–70%)	0	0	0	0	0	0	0	0	0	0	0	2	1
(70%–100%)	0	0	0	0	0	0	0	0	0	0	0	0	0

## Discussion

Predictive models developed in a study where treatment regimens are allocated according to patient characteristics and prognostic factors will likely lead to poor performance when applied to patients who may be treated differently. The extent of bias depends on the proportion of treated patients and the treatment effect on both the primary outcome and competing events. In this study we applied the g-formula to predict metastasis risk in the TPCP cohort of prostate cancer patients under the hypothetical treatment scenario where no patients underwent prostatectomy. Updating the MSKCC model in this counterfactual cohort improved its performance only marginally compared to applying the MSKCC model developed in the observed cohort to the counterfactual cohort. We expect larger differences when the treatment is more strongly associated with the event of interest. Notably, the risk of metastasis in the counterfactual cohort was also influenced by the competing risk of death from other causes. The predicted probabilities of the outcome of interest under counterfactual treatment strategies change not only due to the treatment itself, but also based on the magnitude of its effect on the competing event. For simplicity, we defined prostatectomy as the only treatment of interest and did not include other interventions that may have comparable or smaller, but still meaningful, effects. Additionally, we assumed that surgery was not the primary treatment if performed after six months from diagnosis. Note that, as the landscape of prostate cancer treatment is evolving, and the TPCP cohort reflects the treatment approaches of patients diagnosed around 15 years ago, the predicted progression under the counterfactual treatment scenario, i.e., where no patients underwent prostatectomy, may not be applicable to current patients. Further, we focused exclusively on prostatectomy because the clinical indications for radiotherapy are heterogeneous, encompassing first-line treatment with curative intent, treatment for patients who are not eligible for surgery, and palliative care. As we could not reliably distinguish among these indications, and because patients who did not undergo prostatectomy as first-line treatment may have received a range of alternative management strategies—including active surveillance, watchful waiting, hormone therapy, or radiotherapy—we classified treatment into two categories: prostatectomy and no prostatectomy. However, the methodology used here allows for prediction under different treatment strategies within the same population. Had the goal been to transport counterfactual predictions under different treatment strategies to a different population, additional assumptions would be required (24). Moreover, transportability methods would be needed to be adapted to this framework.

Several studies explored methods for developing and validating predictive models under hypothetical interventions. Lin et al. reviewed 13 papers published between 2015 and 2019, some of them based on combining data from randomised controlled trials with observational data, others using only observational data with different type of outcomes (25). Pajouheshni et al. (5) compared four strategies for developing prognostic models in partially treated cohorts, including: (i) excluding or censoring treated patients, (ii) inverse probability treatment weighting (IPTW) (26–28), (iii) modelling treatment as a time-dependent covariate (29), and (iv) using marginal structural models (30). They highlighted the importance of evaluating IPTW and marginal structural models across different diseases and treatment settings. To avoid issues related to absence of unmeasured confounders or/and misspecification of treatment model, Xu et al. proposed incorporating causal evidence from clinical trials to estimate treatment-naïve risks in time-dependent treatment setting (31). Dickerman et al. applied the g-formula to predict mortality among HIV patients under both static and dynamic counterfactual treatment scenarios, finding that prediction models were systematically mis-calibrated in populations with different treatment strategies–especially under static interventions (32).

In the context of external validation, Pajouheshnia et al. conducted simulation studies to assess the impact of treatment differences between the development and validation sets, particularly for binary outcomes (33). Keogh and Van Geloven extended these approaches to the survival data by modifying validation datasets through artificial censoring and IPTW (34). They demonstrated their method's validity using a simulated external validation dataset and a split sample approach in the real data, but emphasized the need for further research on cross-validation and bootstrapping.

Our approach, based on g-formula, allows for the estimate of treatment effects under counterfactual scenarios using longitudinal data in the presence of competing risks (20, 21). More generally, g-methods, including the g-formula, inverse probability of treatment weighting, and g-estimation of structural nested models, allow for the estimation of counterfactual risks under any static or dynamic treatment regimen by handling time-dependent exposures and confounding (35). The key strength of the g-formula, in this context is that, through Monte Carlo simulation procedures, counterfactual individual trajectories under hypothetical treatment regimens can be generated, allowing for the assessment of the prediction model's robustness to changes in treatment. The parametric g-formula relies on several assumptions. For identifiability in the counterfactual framework, we must assume consistency, exchangeability between treatment groups, and positivity of treatment assignment (35–38). Additionally, the assumptions of no unmeasured confounding, no measurement error and no model misspecification must hold. Because this method involves specifying multiple regression models, violations of these assumptions can substantially impact results. Evaluating model misspecification can be done by comparing the parametric g-formula and the nonparametric estimate of the outcome risk under the natural course (21). Additionally, alternative approaches, such as inverse probability weighted estimators (IPW) or the iterative conditional expectation parametric g-formula, which rely on different underlying assumptions, could be applied. In our study, the close-agreement between non-parametric and the g-formula estimates of the probability of receiving the prostatectomy and the metastatic PCa risk at 5 years supports the lack of misspecification of the models (Supplementary Appendix Figure 4). However, under the counterfactual scenario where no patients were treated, we predicted a similar risk of metastasis and a higher mortality than those obtained in the observed cohort. These findings are explained by the estimated strongly inverse association between prostatectomy and mortality from causes different from prostate cancer, likely due to the selection of the patients eligible for the treatment. Although variables such as age at diagnosis, number of comorbidities and socio-economic status were included in the model for mortality, residual confounding (confounding by indication/selection bias) may have led to models misspecification and, consequently, a violation of one of the core assumptions of the g-formula. However, alternative approaches, such as ignoring treatment, stratifying by treatment, or regressing on treatment, also involve modelling the competing cause of death and are similarly susceptible to bias from model misspecification. Assessing and comparing the extent of bias across these methods may be subject of future work.

Given that we defined treatment as prostatectomy within 6 months of diagnosis, we focused only on static interventions, as treatment choice was independent of disease progression. However this methodology can be readily extended to more complex settings involving time-varying confounding or dynamic treatment strategies. Since only one patient was lost to follow-up, we excluded him for simplicity. However, methods such as the inverse probability of censoring weighting could be adapted for more complex censoring scenarios (18). Finally cause-specific hazard models were preferred to Fine-Gray models for predicting cumulative incidence because they guarantee that the estimated cumulative incidence functions sum to no more than one and can accommodate a wider range of cumulative incidence shapes (15, 39). Notably, our findings were consistent when internal validation via bootstrapping was performed.

## Conclusion

Our findings highlight the need to account for evolving treatment strategies when developing prognostic models. When a prediction model cannot be validated in a new population due to treatment shifts, it is still possible to estimate counterfactual risks under hypothetical treatment regimens–though it requires additional, untestable causal inference assumptions. In a competing risk framework, the predicted risk of the primary outcome is likely to be affected by the predicted risk of competing risk. Thus, even if a hypothetical treatment does not directly affect the counterfactual risk of the outcome, it may still affect predictions through its impact on the competing event. Counterfactual modeling should be considered as a useful complementary assessment of model transportability and calibration under clinically relevant treatment scenarios.

## Data Availability

The datasets presented in this article are not readily available because of legal and ethical reasons. However, to explore the possibility of using the TPCP cohort data for the replication and external validation of a prognostic model for PCa, please contact Lorenzo Richiardi, the Principal Investigator for the TPCP cohort. Although data access may be difficult in the current legal framework, we are open to supporting data reuse at least by discussing an analysis plan, implementing it in our cohort and sharing the results. Requests to access the datasets should be directed to lorenzo.richiardi@unito.it.
