# Supplementary material for: Predicting disease progression under a “treatment-naive” counterfactual scenario in a competing risks framework
Source: Front Epidemiol. 2026 Sep 15;6:1850482. doi: 10.3389/fepid.2026.1850482 (PMC13619617; doi:10.3389/fepid.2026.1850482)
Supplement: Supplementary file 1 [file Datasheet1.docx]

**Supplementary material**

| **Appendix: Table 1**. Descriptive characteristics of the patients of the TPCP cohort included and excluded from the analysis (N=710 and N=181 respectively) | | | | | |
| --- | --- | --- | --- | --- | --- |
|  | N | % | N | % | p-value^ |
| Age at diagnosis (yr), median (IQR) | 70 (64,74) |  | 68 (63,73) |  | 0.07 |
| Calendar year at diagnosis | 2010 (2008,2012) |  | 2010 (2008,2012) |  | <0.001 |
| 2008 | 207 | 29.1 | 79 | 43.6 |  |
| 2009 | 146 | 20.6 | 37 | 20.4 |  |
| 2010 | 58 | 8.2 | 12 | 6.6 |  |
| 2011 | 107 | 15.1 | 30 | 16.6 |  |
| 2012 | 100 | 14.1 | 10 | 5.5 |  |
| 2013 | 92 | 13.0 | 13 | 7.2 |  |
| PSA (ng/ml), median (IQR) (missing, n=23) | 6.7 (5.1,10.2) |  | 6.7(4.7,9.6) |  | 0.53 |
| Clinical tumour stage (missing, n=68) |  |  |  |  |  |
| Ct1c | 312 | 43.9 | 54 | 47.8 | 0.65 |
| cT2 | 249 | 35.1 | 39 | 34.5 |  |
| cT3 | 149 | 21.0 | 20 | 17.7 |  |
| ISUP grade (missing, n=8) |  |  |  |  |  |
| 1 | 84 | 11.8 | 40 | 23.1 | 0.001 |
| 2 | 226 | 31.8 | 61 | 35.3 |  |
| 3 | 136 | 19.1 | 29 | 16.8 |  |
| 4 | 195 | 27.5 | 30 | 17.3 |  |
| 5 | 69 | 9.7 | 13 | 7.5 |  |
| Number of positive cores, median (IQR) (missing, n=56) | 3 (2,5) |  | 3 (1,5) |  | 0.31 |
| Number of negative cores, median (IQR) (missing, n=56) | 11 (8,14) |  | 11 (8,13) |  | 0.73 |
| Number of comorbidities at diagnosis |  |  |  |  |  |
| 0 | 603 | 84.9 | 166 | 91.7 | 0.06 |
| 1 | 31 | 4.4 | 5 | 2.8 |  |
| 2+ | 76 | 10.7 | 10 | 5.5 |  |
| Social deprivation index, median (IQR) | 0.63(-0.48,1.46) |  | 0.41(-0.31,1.36) |  | 0.73 |
| Treatment (within 6 months) |  |  |  |  |  |
| Prostatectomy  Other (radiotherapy, ormonotherapy, palliative therapy) | 302  408 | 42.5  57.5 | 63  118 | 34.8  65.2 | 0.06 |
| Outcome  Metastatic disease  Death from other causes | 48  83 |  | 9  17 |  | 0.34  0.38 |
| IQR, Interquartile range; PSA, Prostate-Specific Antigen; ISUP, International Society of Urological Pathology  ^ : Chi-square test for categorical variables, Kruskal-Wallis test for continuous variables, Gray’s test for cumulative incidences | | | | | |

| **Appendix: Table 2a**. Hazard ratios (HR) and confidence intervals (CI) for progression to metastasis estimated by cause-specific Cox model in the observed cohort (N=709) | | |
| --- | --- | --- |
|  | HR | CI |
| Age at diagnosis (centered-yr) | 1.12 | 0.81-1.55 |
| PSA (ng/ml) | 1.01 | 1.00-1.01 |
| Clinical tumour stage |  |  |
| Ct1c | 1.00 | ref |
| cT2 | 1.79 | 0.74-4.3 |
| cT3+ | 3.01 | 1.24-7.33 |
| ISUP grade |  |  |
| 1 | 1.00 | ref |
| 2 | 0.37 | 0.08-1.67 |
| 3 | 0.26 | 0.04-1.61 |
| 4 | 1.47 | 0.41-5.33 |
| 5 | 3.58 | 0.92-14.00 |
| Number of positive cores | 0.99 | 0.88-1.11 |
| Number of negative cores | 0.92 | 0.85-1.00 |

| **Appendix: Table 2b**. Hazard ratios (HR) and confidence intervals (CI) for death estimated by cause-specific Cox model in the observed cohort (N=709) | | | | | |  |
| --- | --- | --- | --- | --- | --- | --- |
|  | | HR | | CI | |  |
| Age at diagnosis (yr) | | 2.03 | | 1.55-2.66 | |  |
| PSA (ng/ml) | | 1.01 | | 1.00-1.01 | |  |
| Clinical tumour stage | |  | |  | |  |
| Ct1c | | 1.00 | | ref | |  |
| cT2 | | 1.32 | | 0.80-2.18 | |  |
| cT3+ | | 0.76 | | 0.39-1.47 | |  |
| ISUP grade | |  | |  | |  |
| 1 | | 1.00 | | ref | |  |
| 2 | | 2.79 | | 0.83-9.40 | |  |
| 3 | | 2.58 | | 0.74-8.97 | |  |
| 4 | | 2.83 | | 0.83-9.69 | |  |
| 5 | | 3.38 | | 0.89-12.90 | |  |
| Number of positive cores | | 1.02 | | 0.93-1.12 | |  |
| Number of negative cores | | 0.96 | | 0.91-1.02 | |  |
| Calendar year (unit increase) | | 1.05 | | 0.94-1.19 | |  |
| Number of comorbidities at diagnosis | |  | |  | |  |
| 0 | | 1.00 | | ref | |  |
| 1 | | 2.86 | | 1.32-6.17 | |  |
| 2+ | | 3.59 | | 2.18-5.92 | |  |
| Social deprivation index | | 1.05 | | 0.92-1.19 | |  |
| **Appendix: Table 3**. Calibration intercept and calibration slope values among observed cohort and patients who did not receive prostatectomy at different time-points since diagnosis | | | | | | |
|  | **Time since diagnosis** | | | | | |
|  | 3 years | | 4 years | | 5 years | |
|  | **Original cohort** | | | | | |
| **Calibration intercept** | 0.15 (95% CI: -0.24;0.55) | | 0.01 (95% CI: -0.35;0.38) | | 0.00 (95% CI: -0.34;0.33) | |
| **Calibration slope** | 1.26 (95% CI: 0.75;1.78) | | 1.04 (95% CI: 0.66;1.41) | | 1.01 (95% CI: 0.68;1.33) | |
|  | **Subgroup of patients not receiving prostatectomy** | | | | | |
| **Calibration intercept** | 0.25 (95% CI: -0.21,0.71) | | 0.16 (95% CI: -0.26,0.58) | | 0.14 (95% CI:-0.24,0.53) | |
| **Calibration slope** | 1.25 (95% CI:0.62;1.89) | | 1.01 (95% CI:0.57,1.44) | | 1.05 (95% CI:0.64,1.47) | |
|  | | | | | | |
|  | | | | | | |

**Appendix: Figure 1.** Area under the Curve (AUC) estimated in the observed cohort (black solid line) with 95% confidence intervals (black dash line) and optimism-corrected AUC (gray line)

**Appendix: Figure 2**. Calibration plot at 5 years since diagnosis estimated in the observed cohort with 95% confidence interval.

| **Appendix: Table 4a**. Hazard ratios (HR) and confidence intervals (95% CI) for progression to metastasis estimated by cause-specific Cox model in the counterfactual cohort where nobody underwent to treatment (N=7090) | | |
| --- | --- | --- |
|  | HR | 95% CI |
| Age at diagnosis (centered-yr) | 0.97 | 0.89-1.07 |
| PSA (ng/ml) | 1.00 | 1.00-1.01 |
| Clinical tumour stage |  |  |
| Ct1c | 1.00 | ref |
| cT2 | 1.70 | 1.30-2.24 |
| cT3 | 3.06 | 2.34-4.00 |
| ISUP grade |  |  |
| 1 | 1.00 | ref |
| 2 | 0.33 | 0.21-0.53 |
| 3 | 0.25 | 0.15-0.44 |
| 4 | 1.39 | 0.94-2.05 |
| 5 | 3.89 | 2.57-5.87 |
| Number of positive cores | 0.97 | 0.93-1.00 |
| Number of negative cores | 0.91 | 0.89-0.93 |

| **Appendix: Table 4b**. Hazard ratios (HR) and confidence intervals (95% CI) for death estimated by cause-specific Cox model in the counterfactual cohort where nobody underwent to treatment (N=7090) | | |
| --- | --- | --- |
|  | HR | 95% CI |
| Age at diagnosis (yr) | 1.73 | 1.60-1.86 |
| PSA (ng/ml) | 1.01 | 1.00-1.01 |
| Clinical tumour stage |  |  |
| Ct1c | 1.00 | ref |
| cT2 | 1.42 | 1.24-1.63 |
| cT3 | 0.75 | 0.62-0.91 |
| ISUP grade |  |  |
| 1 | 1.00 | ref |
| 2 | 3.98 | 2.74-5.77 |
| 3 | 3.78 | 2.58-5.53 |
| 4 | 4.20 | 2.88-6.13 |
| 5 | 5.02 | 3.33-7.55 |
| Number of positive cores | 1.01 | 0.99-1.04 |
| Number of negative cores | 0.96 | 0.95-0.98 |
| Calendar year (unit increase) | 1.12 | 1.08-1.16 |
| Number of comorbidities at diagnosis |  |  |
| 0 | 1.00 | ref |
| 1 | 3.32 | 2.68-4.10 |
| 2+ | 3.15 | 2.73-3.64 |
| Social deprivation index | 1.04 | 1.01-1.08 |

| **Appendix: Table 5**. Performance measures of the MSKCC nomogram at 5 years since diagnosis with 95% confidence intervals (95% CI) | | | |
| --- | --- | --- | --- |
|  | **AUC** | **Calibration intercept** | **Calibration slope** |
| 1.Model developed in the observed cohort | 0.82 (95% CI: 0.75;0.89) | 0.00 (95% CI: -0.34;0.33) | 1.01 (95% CI: 0.68;1.33) |
| 2.Model developed in the observed cohort and applied to subgroup of untreated patients | 0.83(95% CI:0.74;0.92) | 0.14 (95% CI:-0.24,0.53) | 1.05 (95% CI:0.64,1.47) |
| 3.Applied model in the counterfactual cohort | 0.83 (95% CI: 0.81;0.85) | 0.05 (95% CI: -0.04;0.15) | 1.07 (95% CI: 0.97;1.17) |
| 4.Updated model in the counterfactual cohort | 0.84 (95% CI: 0.82;0.85) | 0.00 (95% CI: -0.10;0.10) | 1.02 (95% CI:0.99;1.12) |
| Confidence intervals of models 1, 2, and 3/4 cannot be directly compared because of difference in sample sizes | | | |

**Appendix: Figure 3.** Calibration plot at 5 years since diagnosis estimated in the counterfactual cohort where nobody underwent to treatment: model updated in the counterfactual cohort (black line) and model developed in the observed cohort and applied to counterfactual cohort (gray line).

| **Appendix: Table 6.** Reclassification table of the predicted risks of mortality from other causes at 5 years since diagnosis in the observed cohort (N=709) by the MSKCC nomogram developed in the observed cohort (applied model) and the MSKCC nomogram updated under the counterfactual scenario in which no patient was treated (updated model). | | | | | | | | | | | | | |
| --- | --- | --- | --- | --- | --- | --- | --- | --- | --- | --- | --- | --- | --- |
|  | **Updated model** | | | | | | | | | | | | |
| **Applied model** | **[0-5%)** | **[5-10%)** | **[10-**  **15%)** | **[15-20%)** | **[20-25%)** | **[25-30%)** | **[30-35%)** | **[35-40%)** | **[40-45%)** | **[45-50%)** | **[50-60%)** | **[60-70%)** | **[70-100%)** |
| **[0-5%)** | 155 | 85 | 1 | 0 | 0 | 0 | 0 | 0 | 0 | 0 | 0 | 0 | 0 |
| **[5-10%)** | 0 | 94 | 103 | 14 | 0 | 0 | 0 | 0 | 0 | 0 | 0 | 0 | 0 |
| **[10-15%)** | 0 | 0 | 40 | 55 | 13 | 0 | 0 | 0 | 0 | 0 | 0 | 0 | 0 |
| **[15-20%)** | 0 | 0 | 0 | 20 | 23 | 13 | 2 | 0 | 0 | 0 | 0 | 0 | 0 |
| **[20-25%)** | 0 | 0 | 0 | 0 | 5 | 11 | 6 | 1 | 0 | 0 | 0 | 0 | 0 |
| **[25-30%)** | 0 | 0 | 0 | 0 | 0 | 2 | 4 | 4 | 2 | 0 | 0 | 0 | 0 |
| **[30-35%)** | 0 | 0 | 0 | 0 | 0 | 0 | 3 | 5 | 4 | 3 | 0 | 0 | 0 |
| **[35-40%)** | 0 | 0 | 0 | 0 | 0 | 0 | 0 | 4 | 4 | 2 | 5 | 0 | 0 |
| **[40-45%)** | 0 | 0 | 0 | 0 | 0 | 0 | 0 | 0 | 1 | 1 | 3 | 1 | 0 |
| **[45-50%)** | 0 | 0 | 0 | 0 | 0 | 0 | 0 | 0 | 0 | 1 | 4 | 3 | 0 |
| **[50-60%)** | 0 | 0 | 0 | 0 | 0 | 0 | 0 | 0 | 0 | 1 | 5 | 4 | 0 |
| **[60-70%)** | 0 | 0 | 0 | 0 | 0 | 0 | 0 | 0 | 0 | 0 | 0 | 3 | 1 |
| **[70-100%)** | 0 | 0 | 0 | 0 | 0 | 0 | 0 | 0 | 0 | 0 | 0 | 0 | 3 |

 **Appendix Figure 4**. Non-parametric estimates (red line) and estimates obtained by g-formula (green line) for probability of receiving prostatectomy (P(A)) (left panel) and cumulative incidence of metastatic prostate cancer (right panel).
